# The Life Quality and Sexual Function of Women Underwent Radical Hysterectomy

**DOI:** 10.31557/APJCP.2021.22.2.581

**Published:** 2021-02

**Authors:** Roza Pak, Tolkyn Sadykova, Dilyara Kaidarova, Murat Gultekin, Gulnara Kasimova, Shynar Tanabayeva, Naylia Ussebayeva, Aigul Tazhiyeva, Maksut Senbekov, Ildar Fakhradiyev

**Affiliations:** 1 *Kazakh Institute of Oncology and Radiology. Almaty, Kazakhstan. *; 2 *S.D. Asfendiyarov Kazakh National Medical University, Almaty, Kazakhstan. *; 3 *Department of Obstetrics and Gynecology, Division of Gynaecological Oncology, Hacettepe, University Faculty of Medicine, Ankara, Turkey. *; 4 *Kazakhstan’s School of Public Health, Almaty, Kazakhstan. *; 5 *Kazakh Medical University of Continuing Education, Almaty, Kazakhstan. *

**Keywords:** Quality of life, sexual function, cervical cancer, radical hysterectomy

## Abstract

**Background:**

Up to date, there no studies were conducted on the quality of life (QL) and sexual function (SF) of women from Kazakhstan treated for cervical cancer. The study was aimed at the assessment of the QL and SF of women of the Kazakh population who underwent radical hysterectomy compared with chemo-radiotherapy group.

**Methods:**

The study was conducted prospectively on 157 women of the Kazakh population. 92 women underwent radical hysterectomy (RH) and 65 underwent chemo-radiotherapy (CRT). The information was collected before treatment (T1), 6 months (T2) and 12 months (T3) after treatment.

**Results:**

The women’s average age was 41.12 ± 5.4 in the RH group and 47.24 ± 6.1 in the CRT group (p = 0.2). We did not detect significant differences between both groups according to the QLQ C-30 questionnaire (T1). The differences between the RH and CRT groups (p≤0,05) were observed in terms of physical functioning, fatigue, nausea and vomiting, pain during the T2 period. High rates of emotional functioning (p = 0.03), global health and QL (p = 0.02), and symptoms of fatigue (p = 0.04) were detected in the RH group compared to the CRT group during T3. However, pain symptoms (p = 0.001), nausea and vomiting and loss of appetite (p = 0.03) were dominated the CRT group. According to the results of FSFI-6 in the RH group, indicators for the domains “desire” (p = 0.02), “excitement” (p = 0.03), and “orgasm” (p = 0.05) were high, unlike in the CRT group during the T3 period. Nevertheless, the number of complains on the ‘pain during intercourse’ in the CRT group was higher than in the RH group (p = 0.001).

**Conclusion:**

Women who underwent RH had better health scores, global health status, and SF compared with patients treated with CRT.

## Introduction

Cervical cancer is the fourth most occurring female malignancy worldwide (Korfage et al., 2009; Sabulei and Maree, 2019). In Kazakhstan, the incidence rate of cervical cancer is fourth amongst all malignant neoplasms. It constitutes around 5.68% of all female cancers, and it is the reason for death in 9.1% of patients with female genital cancers (KazNIOR, 2019). The results of the recent studies indicated that the choice of treatment method of cervical cancer stage I-IIa should not depend on survival rates since. It was also shown that 5-year survival remains at the same level in the case of radical hysterectomy and chemo-radiotherapy (Landoni et al., 1997). 

The choice of a treatment method must be based on the assessment of clinical factors, such as menopausal status, concomitant diseases, histological type and tumour size (Landoni et al., 2017). It was demonstrated that the surgery or chemoradiotherapy can help to treat 80-95% of women at an early stage of the disease (stages I and II) (Petignat and Roy, 2007). However, the early and delayed effects of such treatment might have a significant negative impact on the quality of life and sexual function of women (Korfage et al., 2009; Thapa et al. 2018; Jensen et al. 2004). Therefore, such an impact should be taken into account during the selection of the type of treatment. In cancer management, due to the long-term survival of cervical cancer patients, QL has become a significant indicator (Sabulei and Maree, 2019). SF also refers to an important factor determining the QL of the patients (Thapa et al., 2018). There is growing evidence that a better quality of life (QL) and sexual functions (SF) after surgical treatment are higher compared to chemo-radiation grpup (Dos Santos et al., 2019; Frumovitz et al., 2005).

The classic approach has been predominantly focused on the prevention and treatment of cervical cancer, and little attention has been paid to the QL and SF of women who have received treatment for cervical cancer (Frumovitz et al., 2005). To present, there were no studies on the assessment of the quality of life (QL) and sexual function (SF) of women in the Kazakhstan population after treatment for cervical cancer.

Taking into account above-mentioned facts, the study aimed at the analysis the QL and SF problems of women in the Kazakh population who underwent radical hysterectomy. These data were compared with indicators of chemo-radiotherapy before treatment (T1), 6 months (T2) and 12 months (T3) after the treatment.

## Materials and Methods

The study was based on the assessment of QL and SF of women who underwent cervical cancer treatment. Each case was examined according to a specific algorithm, including evaluation parameters, the clinical indicators and outcome.

The study was approved by the Ethics Committee of the Kazakh Medical University “Higher School of Health” Almaty, Kazakhstan (Local Ethical Commission Approval No. 43 dated by 02.02.2016. All patients received informed consent before inclusion to the study. The procedure for obtaining consent was carried out by directly explaining the goals and objectives of the study to the patient. It also includes the providing the information about the significance of its results for practice, possible risks and complications.

*Participants of the study*

The inclusion of patients into the study groups was carried out prospectively during the period 2016-2019. The groups included the patients aged 18 to 60 years, who were under the supervision at the Joint-Stock company Kazakh Research Institute of Oncology and Radiology, Ministry of Health of the Republic of Kazakhstan, Almaty. The enrolment in study groups was done during counselling patients before starting treatment (chemo-radiation or surgical) for cervical cancer. The inclusion criteria were as follows: informed consent, age over 18, the presence of a verified diagnosis of cervical cancer at stages IA IB, IIA, IIB, and identification of nationality as ethnic Kazakh and absence of other concomitant oncological diseases. The patients had to confirm the existence of sexual life. 

Exclusion criteria were as the followings: recurrent malignant neoplasm, treatment (both radiation and surgery), and disability due to the presence of mental disorders or cognitive impairment.

During the basic interview, the instructions about filling out the questionnaires were explained individually for each respondent.

**Figure 1:**
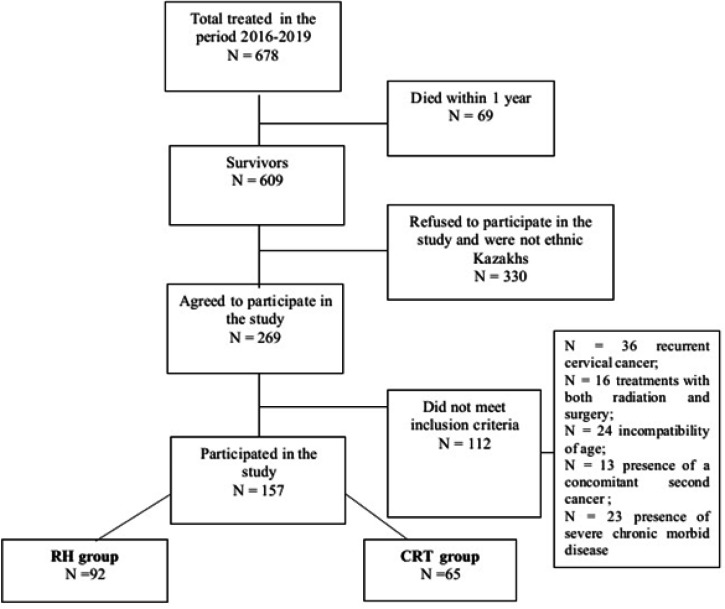
Study Participants

The flow-chart (Figure 1) of participants showed the data of patients treated for the period 2016-2019, where data of 678 patients were used, of which 69 (10.1%) women died within the first year. Among all survivors (n = 609; 89.8 %) nearly 330 patients (55.1 %) refused to participate due to religious reasons, including the questions about sexual function. 

The remaining 269 (44.9 %) patients agreed to participate in the study. However, 112 (41.6 %) did not meet the inclusion criteria due to the following reasons: cervical cancer recurrence, treatment with both radiation and surgery, age, and the presence of a concomitant second cancer or severe chronic morbid disorder. As a result, finally, 157 women were included in the study.

*Study design*

The study groups included patients with a verified diagnosis of cervical cancer before special treatment (surgical or chemo-radiation). Thus, two groups of patients were included in the study:

1st group: radical hysterectomy group (RH) (N = 92), consisted of patients who were treated with a surgical method;

2nd group: chemo-radiotherapy group (CRT) (N = 65), included patients treated with the chemo-radiation;

The study included patients with stages of the disease IA IB, IIA, IIB, IIIA. The standards of International Federation of Gynecology and Obstetrics (FIGO) (Bhatla et al., 2019) were used for the classification of the staging of the tumour process. Socio-demographic data on the age, marital status, presence of children, educational level, as well as the menstrual function of the patients were also evaluated. All methods used in this study were conducted according to the relevant guidelines and rules.

*Evaluation tools*

Two questionnaires were used in this study: a QL questionnaire developed by the European Organization for the Study and Treatment of Cancer (The EORTC quality of life questionnaire (QLQ C-30 v. 3.0) (Fayers et al., 2001), and the multidimensional SF female self-assessment tool The Female Sexual Function Index (FSFI) (Wiegel et al., 2005). We used a validated translated version of the questionnaires in Russian language only (Novitskiy et al., 2009), in order to avoid any language difficulties. 

The QLQ-C30 questionnaire consists of functional and symptomatic scales made of several elements. These include five functional scales (physical, role, emotional, cognitive and social functions), three symptom scales (fatigue, pain, nausea and vomiting), a general health / quality of life rating scale, and six separate points (shortness of breath, insomnia, loss appetite, constipation, diarrhea and financial problems). Each of the scales, consisting of several elements, includes a different set of elements (no element is included in more than one scale). The indicators of all scales and single measurements are in the range from 0 to 100. A high score reflects a higher level of reaction. Thus, a high score for a functional scale shows a high level of functioning (health), and a high score for assessing the state of general health/quality of life reflects a high level of QL. However, a high score for the symptom scale/element reflects a high level of severity of symptoms/health problems (Park et al., 2007). Responses were rated on a Likert scale from “not” to “very strong” or from “very poor” to “excellent”.

A questionnaire for women with a Sexual Function Index-6 (SFI-6) (Filocamo et al., 2014) is a reliable method for identifying symptoms of sexual dysfunction. It allows to distinguish between SFD, with 93% sensitivity and 94% specificity. SF in patients in both groups was evaluated by the following points of the SFI questionnaire: “desire”, “excitement”, “lubrication”, “orgasm”, “satisfaction”, and “pain”. Each subscale is rated from 0 or 1 (worst possible sexual outcome) to 5 (best possible sexual outcome). The total score varies from 3 to 30. A total score of ≤ 19 was considered as an indicator of sexual dysfunction (Isidori et al., 2010) SFI-6 and it was performed independently by each participant in a special ward without any possible influence and / or interference from doctors or other medical personnel. Sexual counselling was offered to women with a total score of SFI-6 ≤ 19. 

QL and SF were evaluated using questionnaire data according to the following schedule: in the preoperative period or before chemo-radiotherapy (T1), 6 months (T2) and 12 months (T3) after treatment.

*Statistical analysis*

The completed questionnaires were placed in a sealed envelope and numbered sequentially. Variables have been described using standard deviation (SD) means and range medians. Data were analysed using SPSS 22.0. Intergroup results were compared using independent t-tests and the Mann-Whitney U test, p values <0.05 were considered statistically significant.

## Results

The main socio-demographic characteristics of the studied groups are presented in Table 1. The average age was 41.12 ± 5.4 in the RH group and 47.24 ± 6.1 in the CRT group without a significant statistical difference (p = 0.2). In the context of marital status, the vast majority of respondents in the RH group and in the CRT group with indicators of 88 % and 66.2 %, respectively, were married. 94.6 % of respondents in the RH group and 96.9 % in the CRT group had one or more children.

In terms of education, the majority of women in the RH group (71.7 %) and in the CRT group (64.6 %) had a secondary education. According to menstrual function, 64.1 % of respondents in the RH group and 69.2 % in the CRT group referred themselves to women with regular menstrual function. About 13 % of the respondents were in menopause. 

In the RH group 62 (67.5%) of respondents were diagnosed with stage IA, and 24 (26%) with stage IB of cervical cancer. At the same time, only 6 (6.5%) of respondents in the RH group were diagnosed with IIA stage. In the CRT group, the smallest number of respondents was treated with the chemo-radiation method in stages IB 2 (3%) and IIA 4 (6%), respectively. The majority of patients 35 (53.9%) from the CRT group were treated according to the protocol for stage IIB and 24 (37%) of the respondents were assigned to the group with IIIA stage of cervical cancer.

Evaluation of QL based on the EORTC QLQ-C30 *Questionnaire*

The results of the assessment of QL in the preoperative period and before chemo-radiotherapy (T1) are presented in Table 2. According to five functional scales, such as physical (p = 0.5), role (p = 0.8), emotional (p = 0.7), cognitive (0.8) and social functioning (p = 0.4) did not show a statistically significant difference between the RH and CRT groups. 

**Figure 2:**
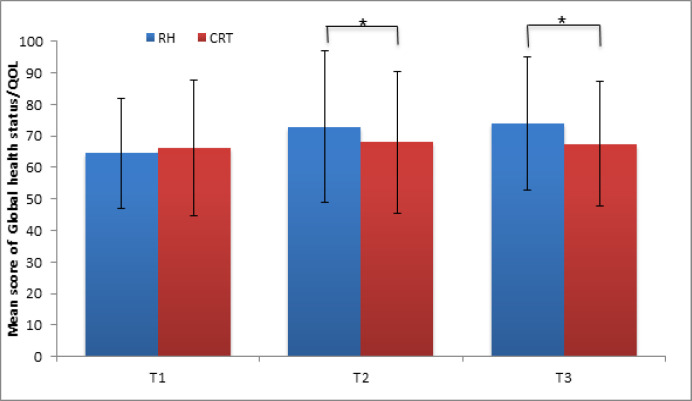
Global Health Status and Quality of Life in RH and CRT Groups (Periods T1, T2 and T3). Data represented as mean ±SD with significance * p <0.05. Assessment of the overall health / quality of life scale since 6 months (T2) after treatment in RH group vs CRT group showed higher patient satisfaction with health and the quality of life (*p = 0.04). After 12 months from the start of treatment RH group respondents vs CRT group continued to note higher satisfaction with their state of health (*p = 0.02). Mann-Whitney test*; T1, in the preoperative period or before chemo-radiotherapy; T2, period of 6 months after treatment; T3, period of 6 months after treatment

There are no statistically significant differences in the fatigue indicators (30.7 ± 24.3 vs. 29.9 ± 21). In addition, we did not detect statistically significant differences of nausea and vomiting (5.1 ± 13.3 vs 5.4 ± 12.0) and the pain scale (31.8 ± 25.1 vs 30.4 ± 24.8) in the RH and CRT groups, respectively (p> 0.05). The results of the assessment of the general health/quality of life in the RH group (64.6 ± 17.4) in comparison with the CRT group (66.3 ± 21.5) did not show a significant difference (p = 0.8) (Figure2). For the remaining six indicators, the loss of appetite was highest in the groups RH (30.6 ± 24.9) and CRT (29.1 ± 17.6), but without any statistical significance (p = 0.5).

The absence of a statistically significant difference for all indicators of this questionnaire shows a uniformity of QL in both groups before treatment.

The data of the repeated QL assessment 6 months after radical hysterectomy and chemo-radiotherapy (T2) are presented in Table 2. According to the data obtained in the T2 period, among the indicators of functional scales, the scores for physical functioning in the RH group (82.3 ± 13.4) were higher compared to the group CRT (76.1 ± 15.8) with a statistically significant difference (p = 0.03).

On the symptom scale, the maximum difference was observed for the symptom of nausea and vomiting that was higher in the CRT group with data of 89.6 ± 12.7 compared with the low scores in the RH group (5.9 ± 11.1) with a marginal statistically significant difference (p = 0.04). The symptom of loss of appetite was disturbed by more respondents of the CRT group (27.5 ± 10.7) compared with the RH group (19.1 ± 18.9) with a statistically significant difference (p = 0.05). The complaint on the pain symptom prevailed in the CRT group, which was 27.5 ± 14.9 (p = 0.04) compared to the RH group (25.3 ± 10.7). At the same time, the symptom of fatigue was more pronounced in the RH group with 29.8 ± 14.8 with a significant difference (p = 0.02). The overall health / quality of life scale showed a significantly higher patient satisfaction with health and the quality of life (p = 0.04) in the RH group (72.9 ± 24.1) compared to the CRT group (68 ± 22.4) (Figure 2) (6 months after the treatment).

According to the assessment of QL, after 12 months (Table 2) following radical hysterectomy and chemo-radiotherapy (T3), a decrease in emotional functioning in the CRT group was observed (74.4 ± 11.4) in comparison with the RH group, where this indicator was 83.3 ± 7.9 (p = 0.03). After 12 months, the symptom of nausea and vomiting was more pronounced for the respondents of the CRT group (9.7 ± 11.2), in contrast to the RH group (5.8 ± 14.2) (p = 0.03). 12 months after treatment, patients in the RH group noticed a significant reduction in pain symptoms, in contrast to the CRT group (17.4 ± 19.9 vs. 24.0 ± 23.3) (p = 0.001). At the same time, complaints on fatigue were lower in the CRT group (25.5 ± 20.4, p = 0.04) in comparison with the RH group (29.9 ± 15.7). Evaluation of the appetite loss scale data demonstrated significantly higher numbers in the CRT group, with an indicator of 24.3 ± 19.6, in contrast to the RH group with scores of 17.2 ± 16.3 (p = 0.03).

After 12 months from the start of the study on the global state of health and quality scale, the RH group respondents continued to note higher satisfaction with their state of health (p = 0.02) in contrast to the CRT group (73.9 ± 21.2 vs. 67.4 ± 19.8) (Figure 2). 

*Results of Self-Assessment for SFI*

**Figure 3:**
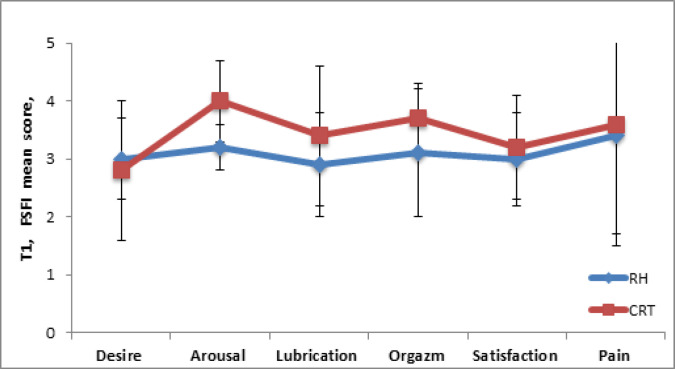
Assessment of the Sexual Function of Women According to the SFI Questionnaire before Treatment (T1) in RH and CRT Groups. Data represented as mean ±SD with significance * p <0.05. Indicators for the symptoms “arousal” (*p = 0.001) and “lubrication” (*p = 0.02) during intercourse were higher in CRT group vs RH group

According to the results of the SFI questionnaire in the T1 period (Figure 3) in the RH and CRT groups, the total score of sexual function was 19.1 ± 4.1 and 20.9 ± 4.8, respectively, without statistical significance (p = 0.2). In the CRT group, the indicators for the symptom “excitation” (p = 0.001) and “lubrication” (p = 0.02) during intercourse were 4.0 ± 0.7 and 3.4 ± 1.2. These figures were higher than the results in RH group, with a statistically significant difference.

**Figure 4:**
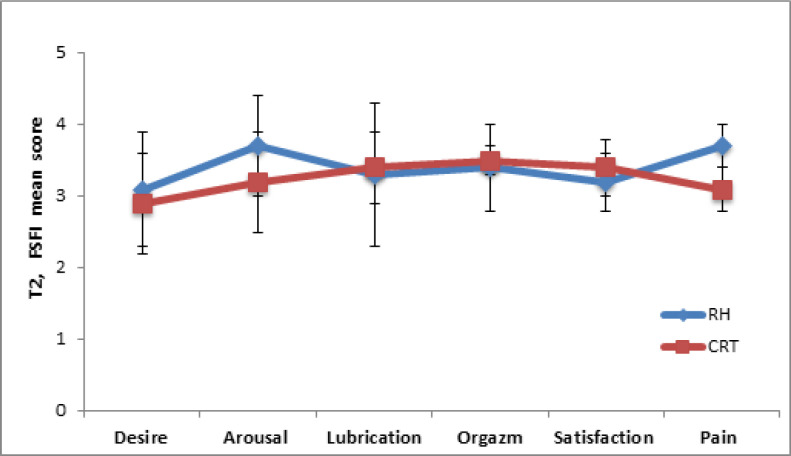
Assessment of the Sexual Function of Women According to the SFI Questionnaire since 6 Months (T2) after Treatment in RH and CRT Groups. Data represented as mean ±SD with significance * p <0.05. The level of “arousal” of patients of was lower in the CRT group vs RH group (*p = 0.04), indicator of “pain” during intercourse was bigger in CRT group vs RH group (*p = 0.001).

RH respondents noted a higher level of “arousal” during sexual intercourse (3.7 ± 0.7) 6 months after treatment (T2) (Figure 4). At the same time, the level of “arousal” of patients of the CRT was 3.2 ± 0.7 group (p = 0.04).

The indicators of the absence of sensation of “pain” during or after intercourse were lower in the CRT group (3.1 ± 0.3) than in the RH group (3.7 ± 0.3) with a statistically significant significance (p = 0.001). For the remaining SF factors, there were no statistically significant differences between the two groups (6 months after the start of treatment).

**Figure 5:**
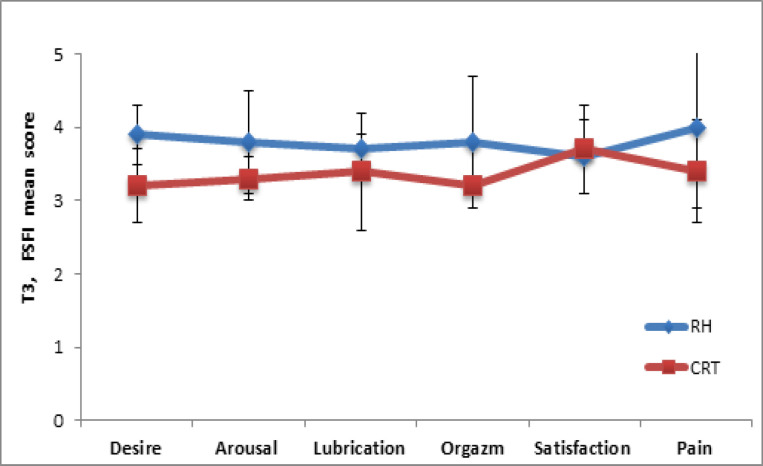
Assessment of the Sexual Function of Women According to the SFI Questionnaire Since 12 Months (T3) after Treatment in RH and CRT Groups. The high indices of “arousal” (*p = 0.03), “desire” (*p = 0.02) and “orgasm”(*p = 0.05) were registered in RH group vs CRT group, “pain” symptom was dominated in CRT group vs RH group (*p = 0.001).

After 12 months (T3), the results of the SFI questionnaire indicated (Figure 5) that for the symptoms “lubrication” (p = 0.8) and “satisfaction” (p =0.6), there were no statistically significant differences in both groups (during sexual intercourse). In terms of the “excitation” index, high indices in the RH group also remained unlike for the CRT group (3.8 ± 0.7 vs. 3.3 ± 0.3) with statistical significance p = 0.03.

In contrast to the intervals T1 and T2, 12 months after the therapeutic intervention, the indicators of the “desire” and “orgasm” in the RH group (3.9 ± 0.4 and 3.8 ± 0.9, respectively) were higher in comparison with the CRT group (p = 0.02 and p = 0.05, respectively). It should be noted that complaints about the “pain” symptom during and after coitus were pronounced among the respondents of the CRT group (3.4 ± 0.7), in contrast to the participants in the RH group (4.0 ± 1.1) (p = 0.001). In general, the total SF numbers with a score of 23.5 ± 4.2 in the RH group was higher compared to the CRT group (20.7 ± 3.4) with a statistically significant difference (p = 0.02).

**Table 1:** Characteristics of Participants Depending on the Study Group

	Radical hysterectomy (RH) N (%)	Chemo-radiotherapy (CRT) N, (%)	Total	P values
Age (years)	41.12 ± 5,4	47.24 ± 6.1		0.2
Marital Status				
Unmarried	9 (9.9)	22 (33.8)	31 (21.8)	<0.001
Married	81 (88)	43 (66.2)	124 (77.1)	<0.001
Widow	2 (2.1)	0 (0)	2 (1.05)	<0.001
Children				
None	5 (5.4)	2 (3.1)	7 (4.25)	0,4
One or more	87 (94.6)	63 (96.9)	150 (95.7)	0,4
Education (years)				
Higher	21 (22.8)	13 (20)	34 (21.4)	0.1
Middle	66 (71.7)	42 (64.6)	108 (68.1)	0.1
None	5 (5.4)	10 (15,4)	15 (10,4)	0.1
Menstruation				
regular	59 (64.1)	45 (69.2)	104 (66.6)	<0.001
non regular	19 (20.6)	13 (20)	32 (20.3)	<0.001
menopause	14 (15.2)	79 (10.7)	93 (13)	<0.001
Stage of disease				
IA	62 (67.5%)	0 (0)	62 (67.5%)	<0.001
IB	24 (26%)	2 (3%)	24 (29%)	
IIA	6 (6.5%)	4 (6.1%)	10 (12.6%)	
IIB	0 (0)	35 (53.9%)	35 (53.9%)	
IIIA,В	0 (0)	24 (37%)	24 (37%)	

Values are presented as mean ± standard deviation or number (%).

**Table 2:** Assessment of the Quality of Life (EORTC QLQ-C30 in RH and CRT Groups (Periods T1, T2 and T3)

Period Scale	T1			T2			T3		
	Radical hysterectomy	Chemo- radiotherapy	P	Radical hysterectomy	Chemo- radiotherapy	P	Radical hysterectomy	Chemo- radiotherapy	P
Physical functioning	86.1	81.7	0.5	82.3	76.1	0.03*	81.1	80.8	0.9
Role functioning	84.3	85.2	0.8	86.6	87.4	0.7	88.5	88.3	0.9
Emotional functioning	75.6	77.9	0.7	77.2	76.9	0.07	83.3	74.4	0.03*
Cognitive functioning	88.8	87.4	0.8	86.1	84.3	0.6	88.7	87.5	0.7
Social functioning	86.9	87.1	0.4	87.6	85.5	0.8	87.3	86.2	0.3
Fatigue	30.7	29.9	0.1	29.8	26.7	0.02*	29.9	25.5	0.04*
Nausea and vomiting	5.1	5.4	0.6	5.9	8.6	0.04*	5.8	9.7	0.03*
Pain	31.8	30.4	0.7	25.3	27.5	0.04*	17.4	24	0.001*
Dyspnea	13.2	13.5	0.4	13.4	12.2	0.6	14.1	13.1	0.1
Insomnia	27.4	24.4	0.07	21.1	22.2	0.6	20	21.1	0.5
Loss of appetite	30.6	29.1	0.5	19.1	27.5	0.05*	17.2	24.3	0.03*
Constipation	17.2	16.8	0.8	18.3	16.9	0.2	19.4	17.2	0.2
Diarrhea	9.7	8.8	0.6	16.7	18.3	0.1	15.3	13.5	0.2
Financial difficulties	21.9	20.3	0.3	20.9	22.4	0.09	23.1	20.9	0.3
Global Health and Quality of Life	64.6	66.3±21.5	0.8	72.9	68	0.04*	73.9±21.2	67.4±19.8	0.02*

Mann-Whitney test*; T1, in the preoperative period or before chemo-radiotherapy; T2, period of 6 months after treatment; T3, period of 6 months after treatment.

## Discussion

The objective of this study was to determine the level of QL and SF in women who underwent radical hysterectomy and chemo-radiotherapy at the early stage of cervical cancer. In fact, such studies have never been conducted in the Kazakhstan as well as in Central Asia. The results demonstrated that one year after treatment, satisfaction with QL and SF was significantly higher in the group of respondents who underwent a radical hysterectomy. 

It has been thought that a radical hysterectomy possesses equal therapeutic efficacy to chemo-radiotherapy. In this regard, the choice of surgical intervention or chemo-radiotherapy has been usually dictated by clinical factors such as the stage of the tumour process, oncological risk, problems with anaesthesia and the surgeon’s skills, and women’s personal preferences (Brucker and Ulrich, 2016). At present, radical hysterectomy or chemo-radiotherapy are the main methods for the treatment of cervical cancer at stages Ia – IIa according to the recommendations of the International Federation of Gynecology and Obstetrics (FIGO) (Verleye et al., 2009). It must be noted that both approaches surgery and chemo-radiotherapy for the treatment of cervical cancer have some advantages, limitations and undesirable consequences as well (Wallin et al., 2019). 

According to the results acquired, it can be noted that the use of radical surgery for cervical cancer demonstrated a better level of QL, in particular, higher emotional functioning (p = 0.02) compared with chemo-radiotherapy. 

Even though more than 200 years have passed since the first radical hysterectomy was performed (Verleye et al., 2009), various modifications of this method are aimed at reducing the risk of postoperative complications were proposed (Wallin et al., 2019; Zakashansky et al., 2008; Swailes et al., 2017). In any case, the traditional technique for performing radical hysterectomy has proven its effectiveness over time. According to the data of published reports, some problems and organ dysfunctions after radical hysterectomy might affect the sexual function (Harding et al., 2014). These findings were confirmed by the results of our study. Particularly, we observed the reliably high rates for the factors related to arousal and orgasm in the RH group at 6 and 12 months after surgery (p <0.05). 

The second factor is the toxicity of chemotherapy and radiation therapy that remains a huge issue in oncology (Dueñas-González et al., 2016). The chemo-radiotherapy is preferable method if a tumour size is more than 4 cm or there are contraindications for surgical intervention (Aredes et al., 2018). The optimal approach of the treatment (according to the results of randomized trials) is a combination of radiation therapy and cisplatin or 5-fluorouracil (5-FU) (Eifel, 2006). However, cisplatin possesses severe toxic side effects such as dose-dependent nephrotoxicity that limits its wide application in the clinics. Apart from cisplatin, there is a range of FDA-approved drugs for the treatment of cervical cancer, including avastin, bleomycin sulfate, hycamtin, keytruda, mvasi, pembrolizumab and topotecan hydrochloride. Despite the proven anti-cancer effectiveness, all the above-mentioned compounds possess a various side effects such as high blood pressure, bleeding, inflammation, pain syndrome, etc. 

In fact, chemotherapy can potentiate the effect of radiation treatment thus providing a high efficiency against tumour cells. However, the combination of these two methods leads to the destruction of not only tumour cells, but also healthy cells and the increase in toxicity (Rose et al., 1999). Moreover, such a combination can provoke synergetic toxic effects that can considerably lower QL and SF of the patients. Toxicity can manifest through disorders of the gastrointestinal tract (Vale et al., 2008) accompanied by nausea, vomiting, and diarrhoea (Aredes et al., 2018). These findings were confirmed in our study by an increase of loss of appetite, nausea and vomiting in the CRT group after 6 months. However, these symptoms continued to exist compared to the RH group ‘one year after treatment’.

Sun et al., (2020) studied the QL after applying a radical hysterectomy (compared with chemo-radiation treatment). The statistically significant differences were recorded only for indicators of social functioning and satisfaction with sexual life in the surgical treatment group (2-3 years after treatment). In contrast to the results of our study, no significant differences between experimental groups were found out. 

Another study came to the conclusion that the global health status of groups considerably improved after 12 months followed radiation therapy (Sabulei and Maree, 2019). However, according to the results of our study of the Kazakhstan population, health satisfaction in the RH group remained higher after 6 and 12 months after treatment in comparison with the CRT group.

According to the available published data, the patients diagnosed with cancer are suffering from physical and mental fatigue in comparison with the control group (within 10-15 years after treatment) (Gernier et al., 2020). In another study on the presence of chronic fatigue syndrome (based on the Fatigue Questionnaire) in 23 % of survivors of cervical cancer, this pathological condition was more pronounced in patients treated with the chemo-radiative method compared with patients underwent radical surgery (11 years after treatment) (Steen et al., 2017).

However, the results of the study based on the QL assessment questionnaire, the RH group respondents at 6 months (p = 0.023) and one year (p = 0.023) after treatment noted a prevalence of the fatigue symptom compared to CRT group.

In fact, there is a correlation between QL and problems with reproductive function (Carter et al., 2010a). For instance, a malignant neoplasm of young women is a factor in the occurrence of depression due to the risk of infertility (Carter et al., 2010b). In fact, most women who were diagnosed with cervical cancer can live a long life, however, they can experience mental and social problems due to fertility issues. The choice for cancer patients who receive radio-chemotherapy and who want to maintain fertility mainly relies on clinically established methods such as cryopreservation of embryos or ovarian tissue (Duffy and Allen, 2009). The prevention of fertility problems in women diagnosed with cervical cancer is a significant issue for the healthcare system. However, the inability to solve such issues might have long-term negative consequences for a woman’s QL (Duffy and Allen, 2009). This area requires more attention and relevant doctors’ training in order to optimize the fertility preservation.

Since women in our study in the RH group were over 40 years old and 94.6% had one or more children, it can be assumed that the termination of fertile function after radical surgery did not significantly reduce their quality of life. It is known that radical hysterectomy can induce the post-castration syndrome due to a decrease in the level of oestradiol in the blood. A similar situation can occur as a result of chemotherapy leading to chemical menopause. All this can manifest itself with common menopausal symptoms causing a decrease in sexual function and cognitive abilities (Vearncombe and Pachana, 2009; Verghese et al., 2000). Nevertheless, in our study, the effect of surgical menopause on cognitive function was not detected (Vearncombe and Pachana, 2009).

According to the results of the respondents’ assessment of the SFI questionnaire, one can notice a significant reliable gradual improvement of such indicators as a “desire” (p = 0.02), “arousal” (p = 0.03), and “orgasm achievement” during intercourse (p = 0.05). In general, we observed a high total score (p = 0.02) in the RH group compared to the CRT group (period: from 6 months to 1 year after treatment). According to the results a previous study, difficulties with orgasm, dyspareunia, sexual dissatisfaction, and narrowing of the vagina are short-term adverse effects that persist from 5 to 6 months after surgery (Jensen et al. 2004).

The results of the study carried out by Schover et al.(1989) demonstrated that one year after undergoing radiation therapy, women’s sexual function underwent a significant deterioration compared to the women treated by the surgical method. These data conform to the data of our study. Interestingly, the respondents of the CRT group according to the indicators “excitation” and “lubrication” had statistically significantly higher rates than in the second group before the chemo-radiotherapy treatment. However, after 6 and 12 months the scores for these indicators were low (p <0.05). The symptom of discomfort due to “pain” during intercourse in the radiotherapy group that bothered patients after 6 months (p = 0.001) and 12 months (p = 0.001) can be explained by more severe side effects caused by the radiation (Ye et al., 2014).

An adverse reaction to radiation therapy triggers chronic fibrotic changes in the pelvic tissue and atrophy of the vaginal tissue. In turn, it can cause complaints of dryness, shortening, narrowing of the vagina, and discomfort during intercourse. Some researchers strongly recommended using a vaginal lubricator as a preventive measure for the maintenance of the elasticity of the vaginal wall (Frumovitz et al., 2005). The elimination of unwanted complaints of the patient and improving a balance of QL and SF are important tasks for the physicians (Fernandes and Kimura, 2010).

In conclusion, cervical cancer and its treatment have a negative impact on the quality of life in all areas of the life of women affected by this disorder. At present, the sexual function and quality of life of women (in Kazakhstan) who underwent treatment for cervical cancer remains low. The results of our study indicated that the reduced quality of life indicators in the group of patients after chemo-radiotherapy requires an adequate rehabilitation measure in the early and long-term period following the treatment. In this regard, there is a need to develop a program to improve the quality of life and sexual dysfunction in women who underwent treatment for cervical cancer in the Republic of Kazakhstan.
